# The Relation of Calculated Plasma Volume Status to Sublingual Microcirculatory Blood Flow and Organ Injury

**DOI:** 10.3390/jpm13071085

**Published:** 2023-06-30

**Authors:** Eleni Laou, Nikolaos Papagiannakis, Nicoletta Ntalarizou, Theodora Choratta, Zacharoula Angelopoulou, Konstantinos Annousis, Minas Sakellakis, Aikaterini Kyriakaki, Dimitrios Ragias, Anastasia Michou, Athanasios Chalkias

**Affiliations:** 1 Department of Anesthesiology, Agia Sophia Children’s Hospital, 11527 Athens, Greece; 2 First Department of Neurology, Eginition University Hospital, Medical School, National and Kapodistrian University of Athens, 15772 Athens, Greece; 3 Department of Anesthesiology, Faculty of Medicine, University of Thessaly, 41500 Larisa, Greece; 4 First Department of Surgery, Metaxa Cancer Hospital, 18537 Piraeus, Greece; 5 Department of Emergency Medicine, Tzaneio General Hospital, 18543 Piraeus, Greece; 6 Department of Medical Oncology, Metropolitan Hospital, 10461 Piraeus, Greece; 7 High Dependency Unit, General Hospital of Syros Vardakeio and Proio, 84100 Syros, Greece; 8 Institute for Translational Medicine and Therapeutics, School of Medicine, University of Pennsylvania Perelman, Philadelphia, PA 19104, USA; 9 Outcomes Research Consortium, Cleveland, OH 44195, USA

**Keywords:** calculated plasma volume status, microcirculation, organ injury

## Abstract

Background: The calculated plasma volume status (cPVS) was validated as a surrogate of intravascular filling. The aim of this study is to assess the cPVS in relation to sublingual perfusion and organ injury. Methods: Pre- and postoperative cPVS were obtained by determining the actual and ideal plasma volume levels in surgical patients. The sublingual microcirculation was assessed using SDF imaging, and we determined the De Backer score, the Consensus Proportion of Perfused Vessels (Consensus PPV), and the Consensus PPV (small). Our primary outcome was the assessment of the distribution of cPVS and its association with intraoperative sublingual microcirculation and postoperative complications. Results: The median pre- and postoperative cPVS were −7.25% (IQR −14.29–−1.88) and −0.4% (IQR −5.43–6.06), respectively (*p* < 0.001). The mean intraoperative administered fluid volume was 2.5 ± 2.5 L (1.14 L h^−1^). No statistically significant correlation was observed between the pre- or postoperative cPVS and sublingual microcirculation variables. Higher preoperative (OR = 1.04, *p* = 0.098) and postoperative cPVS (OR = 1.057, *p* = 0.029) were associated with postoperative organ injury and complications (sepsis (30%), anemia (24%), respiratory failure (13%), acute kidney injury (6%), hypotension (6%), stroke (3%)). Conclusions: The calculated PVS was associated with an increased risk of organ injury and complications in this cohort.

## 1. Introduction

The optimization of intravascular volume is important for improving the outcomes of medical or surgical patients. After decades of research, the consequences of hypo- and hypervolemia are well known and potentially serious, and fluid management is a key component of clinical practice. However, fluid imbalance remains a common cause of morbidity and mortality [1]. 

The accurate assessment of fluid status can be difficult, and advanced monitoring may be challenging or unavailable due to practical constraints and/or high costs. Some studies advocate for the use of non-invasive monitoring or sophisticated devices to optimize the assessment of intravascular volume status, but the error percentage of these methods remains considerable. Thus, a reliable and easy-to-use marker of volume status that could be incorporated into the risk stratification algorithm and optimization of a patient’s physiology would be of great value in advanced planning.

Plasma volume is closely associated with the weight and hematocrit value and can be assessed via the application of simple equations. In particular, the calculation of the relative plasma volume status (cPVS) can reveal the degree to which patients have deviated from their ideal plasma volume status [2,3]. Of note, the cPVS was validated as a surrogate of intravascular filling in patients with cardiac disease, its values are correlated with those measured using tracer-dilution assays, and it was associated with mortality in several populations [2,3,4,5,6,7]. 

Monitoring the cPVS could assist in the timely recognition of individual fluid needs or fluid intolerance. It is also possible that changes in the cPVS may be associated with microcirculatory flow alterations and outcomes [8]. Therefore, we hypothesized that the cPVS could serve as a novel tool for perioperative fluid balance monitoring, with prognostic significance. To generate preliminary evidence surrounding this hypothesis, we leveraged a prospective cohort of patients who underwent major non-cardiac surgery, in order to assess the cPVS in relation to sublingual microcirculation and organ injury.

## 2. Materials and Methods

We conducted a post hoc secondary analysis of a prospective observational study at the University Hospital of Larisa, Greece. The primary study was conducted in accordance with Good Clinical Practice guidelines, the principles of the Declaration of Helsinki, and relevant regulatory requirements. The original study was registered in ClinicalTrials.gov (NCT03851965; 22 February 2019) [9]. The University Hospital of Larisa Institutional Review Board approved the study (IRB No. 60580, 11 December 2018). Written informed consent was obtained from each participant or their next of kin.

### 2.1. Study Objective

The primary objective is to investigate the association of cPVS with intraoperative sublingual microcirculation and organ injury after major non-cardiac surgery.

### 2.2. Patient Eligibility 

The protocol was described in detail elsewhere [9]. In brief, we considered adults scheduled for elective major non-cardiac surgery with an expected duration of ≥2 h under general anesthesia. Patients were American Society of Anesthesiologists (ASA) physical status I to IV, and all operative approaches were eligible, including open and laparoscopic procedures. 

We excluded patients who had infections within the previous month; severe liver disease; a need for renal replacement therapy; allergies; inflammatory or immune system disorders; asthma; obesity (BMI ≥ 30 kg m^−2^); mental disability or severe psychiatric disease; alcohol abuse; and connective tissue diseases including rheumatoid arthritis, ankylosing spondylitis, and systemic lupus erythematosus. We also excluded patients who previously received an organ transplant; who were treated with steroids, anti-psychotic, or anti-inflammatory/immunomodulatory medication within the previous three months or with opioids during the past week; and those who were involved in another study [9]. 

### 2.3. Anesthetic Management

Before anesthesia induction, patients were given 5 mL kg^−1^ of a balanced crystalloid solution to compensate for preoperative fasting and anesthetic-induced vasodilation [9,10]. Anesthesia was induced in the supine position and included 0.15–0.35 mg kg^−1^ of midazolam, 1 μg kg^−1^ of fentanyl, 0.2 mg kg^−1^ of ketamine, 1.5–2 mg kg^−1^ of propofol, 0.6 mg kg^−1^ of rocuronium, and a fraction of inspired oxygen of 0.7. After tracheal intubation, patients were mechanically ventilated using a lung-protective strategy with a tidal volume of 7 mL kg^−1^, positive end-expiratory pressure of 6–8 cmH_2_O, and plateau pressure of <30 cmH_2_O. 

General anesthesia was maintained by inhalation of desflurane at an initial 1.0 minimal alveolar concentration. Thereafter, depth of anesthesia was adjusted to maintain Bispectral Index (BIS, Covidien, Paris, France) between 40 and 60. The intraoperative fraction of inspired oxygen was then adjusted to maintain an arterial oxygen partial pressure between 80 and 100 mmHg, and the respiratory rate was adjusted to maintain normocapnia. Normothermia (37 °C core temperature) and normoglycemia were maintained during the perioperative period. Vasopressors were administered when mean arterial pressure (MAP) was < 65 mmHg and their dosage was titrated to maintain an individualized MAP level based on the patient’s preadmission ambulatory/nocturnal levels. The choice of vasopressor was at the discretion of the attending anesthesiologists.

Balanced crystalloids were given at a rate of 2 mL kg^−1^ h^−1^. Surgery-related blood losses were compensated by infusing balanced crystalloids (2:1 ratio) or 6% hydroxyethyl starch 130/0.4 (1:1 ratio). Packed red blood cells were transfused whenever the hemoglobin concentrations were <9–10 g dL^−1^ in patients with cardiovascular comorbidities and the elderly, or <8 g dL^−1^ in those without cardiac comorbidities.

### 2.4. Measurements

During surgery, MAP was directly measured using a 20-gauge radial arterial catheter connected to the anesthesia monitor. Before study measurements, we confirmed that the transducers were correctly leveled and zeroed. We also confirmed the system’s dynamic response, and artifacts were assessed and managed as previously described [11]. 

Sublingual microcirculation was monitored using SDF+ imaging (Microscan; Microvision Medical BV, Amsterdam, the Netherlands). The first assessment was performed 30 min after the induction of general anesthesia before surgical incision. Thereafter, assessments were performed every 30 min until emergence from anesthesia. At each measurement point, sublingual microcirculation videos from at least five sites were recorded. To optimize video quality, we tried to avoid pressure and movement artefacts, optimized focus and illumination, and cleaned saliva and/or blood from the sublingual mucosa. Before analysis, all sublingual perfusion videos were evaluated by two experienced raters blinded to all patient data, according to a modified microcirculation image quality score (MIQS) [12]. The best three videos from each recording were analyzed offline by a blinded investigator using the AVA4.3C Research Software (Microvision Medical, Amsterdam, the Netherlands) [11,13,14]. We analyzed the De Backer score as the density score, and the Consensus Proportion of Perfused Vessels (Consensus PPV), and Consensus PPV (small) as flow scores.

Pre- and postoperative cPVS were obtained by determining the actual and ideal plasma volume levels as previously reported in [2,3,15] as follows:

(1) $Actual\;plasma\;volume=\left(\left[1-hematocrit\right]\times \left[a+\left(b\;\times \;weight\left[\mathrm{kg}\right]\right)\right]\right)$, where *a* and *b* are sex-related constants (*a* = 1530 in males and 864 in females; *b* = 41.0 in males and 47.9 in females).
(2) $Ideal\;plasma\;volume=c\times weight\left(\mathrm{kg}\right)$, where *c* is sex-related constant (*c* = 39 in males and 40 in females).
(3)
$cPVS\left(\%\right)=\frac{Actual\;plasma\;volume\;-\;Ideal\;plasma\;volume}{Ideal\;plasma\;volume}\times 100$

The equation of cPVS correlates with plasma volume estimated using a radiolabeled albumin assay [16]. The cPVS is expressed as a percentage of difference from ideal plasma volume [17]. For example, cPVS of 25 represents an actual plasma volume that is 25% higher than the ideal volume [18]. 

### 2.5. Data Collection, Monitoring, and Management

Data analysis was based on predefined and contemporaneously recorded measurements. Data collection included demographic and morphometric characteristics, ASA physical status, risk scores (Modified Frailty Index, POSSUM risk score, ACS-NSQIP), and anesthesia variables. We also used the Clavien–Dindo Classification and the Comprehensive Complication Index (CCI) to assess postoperative complications, morbidity, and mortality in our patients. Remote monitoring was performed to signal early aberrant patterns, issues with consistency, credibility, and other anomalies. Any missing and outlier data values were individually revised and completed or corrected whenever possible. This work is reported according to STROCSS criteria [19]. 

### 2.6. Statistical Analysis

The Shapiro–Wilk method for testing the normality of data was used to assess the distribution of the various variables. Therefore, non-parametric tests were used. Correlations were computed through Spearman’s method, and Wilcoxon sign rank test was used to examine the paired differences. Logistic regression models were constructed to assess whether PVS was associated with the presence of complications. The Benjamini–Hochberg false discovery rate correction was utilized to adjust for multiple comparisons. A threshold of 0.05 for significance was applied to *p*-values.

## 3. Results

**Figure 1:**
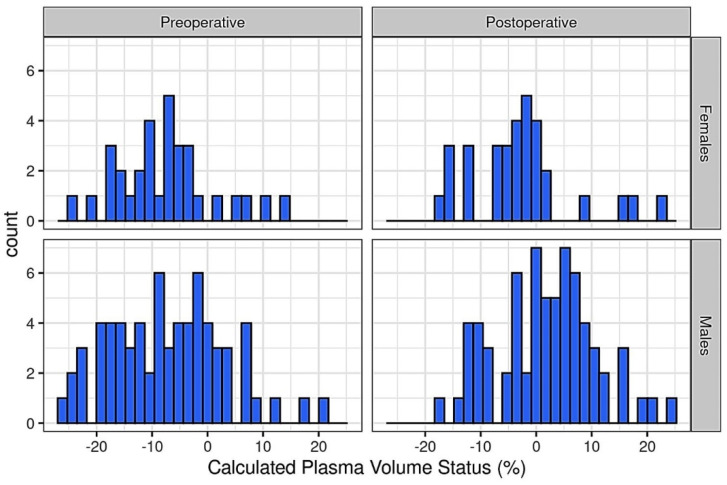
Histogram of pre- and postoperative calculated plasma volume status.

One hundred patients (median age of 70 (IQR 62.8–75.2); males n = 68, females n = 32) were included and assigned to different ASA categories (17 ASA II, 43 ASA III, 40 ASA IV) (Table 1 and Table S1) [9]. The median preoperative cPVS was −7.25% (IQR −14.29–−1.88). The intraoperative fluid administration (2.5 ± 2.5 L (1.14 L h^−1^)) increased the cPVS to −0.4% (IQR −5.43–6.06) by the end of the surgery (*p* < 0.001). The distribution of perioperative cPVS is depicted in Figure 1. There was no statistically significant difference in the cPVS between the male and female patients (*p* = 0.077). Ten (10) patients were transfused with a unit of packed red blood cells during surgery.

A statistically significant correlation was observed between the preoperative cPVS and POSSUM score (morbidity: rho = 0.422, *p* < 0.001; mortality: rho = 0.418, *p* < 0.001), suPAR (rho = 0.268, *p* = 0.007), prothrombin time (rho = 0.332, *p* = 0.004), international normalized ratio (rho = 0.333, *p* = 0.004), and hemoglobin levels (rho = −0.838, *p* < 0.001) (Table S2). The postoperative cPVS was significantly correlated with the POSSUM score (morbidity: rho = 0.347, *p* = 0.004; mortality: rho = 0.340, *p* = 0.004), hemoglobin (rho = −0.745, *p* < 0.001), BMI (rho = −0.554, *p* < 0.001), aPTT (rho = 0.258, *p* = 0.034), C-reactive protein (CRP) (rho = 0.246, *p* = 0.046), total protein (rho = −0.321, *p* = 0.008), and albumin levels (rho = −0.294, *p* = 0.014) (Table S3). 

**Figure 2:**
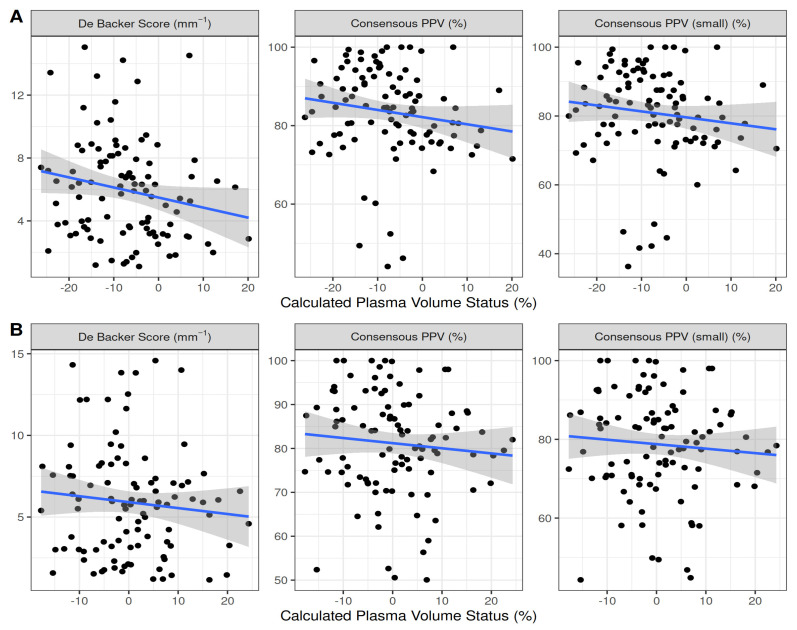
Scatterplot of calculated plasma volume status values and microcirculation variables pre- (**A**) and postoperatively (**B**).

No statistically significant correlation was observed between the pre- or postoperative cPVS and sublingual microcirculation variables (Table 2, Figure 2). Unlike the De Backer score (5.95 ± 3.21 vs. 5.89 ± 3.36, *p* = 0.404), the Consensus PPV (83.49 ± 11.5 vs. 81.15 ± 11.8, *p* < 0.001) and Consensus PPV (small) (80.87 ± 13.4 vs. 78.72 ± 13, *p* < 0.001) decreased significantly from the baseline during surgery. 

**Figure 3:**
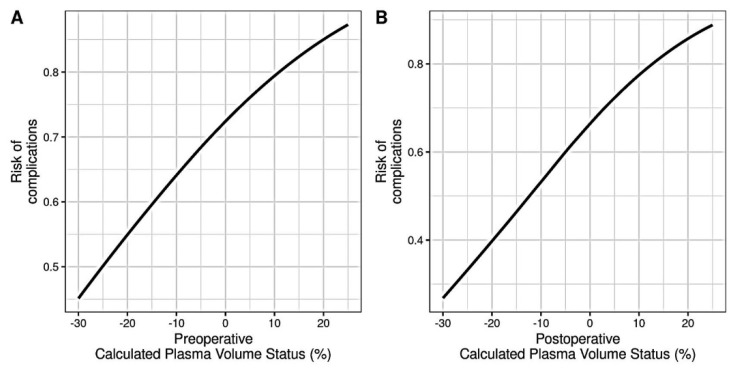
Pre- (**A**) and post- (**B**) operative association of calculated plasma volume status levels with the risk of postoperative complications.

The most frequent complications were sepsis (30%) and anemia (24%), followed by respiratory failure (13%), acute kidney injury (6%), hypotension (6%), and stroke (3%) (Table 3). When accounting for the presence of any complication, a higher preoperative (OR = 1.04, *p* = 0.098) and postoperative cPVS (OR = 1.057, *p* = 0.029) were associated with a higher risk of postoperative organ injury and complications (Figure 3). However, no statistically significant trend was present between the cPVS and the presence of comorbidities (*p* = 0.961). Also, no statistically significant correlation was observed between the pre- to postoperative cPVS difference (ΔcPVS) and postoperative complications (OR = 1.01, *p* = 0.599). Neither the preoperative (rho = 0.174, *p* = 0.230) nor postoperative (rho = 0.16, *p* = 0.210) cPVS was correlated with CCI. 

Within the first 90 postoperative days, five (5%, SE = 2.17%) patients were unexpectedly admitted to the intensive care unit and four (4%, SE = 1.96%) patients died, and the 1-year survival rate was 92% (SE = 2.71%). 

## 4. Discussion

In this post hoc secondary analysis, the preoperative cPVS was −7.25% and was significantly increased to −0.4% by the end of the surgery. However, no significant correlation was observed between the perioperative cPVS and intraoperative sublingual microcirculation variables. The perioperative cPVS, but not its trend, was associated with an increased risk of postoperative organ injury and complications, the most frequent of which were sepsis and anemia, followed by respiratory failure, acute kidney injury, hypotension, and stroke. 

Although preoperative fluid management typically aims to ensure normovolemia and adequate hydration, generous amounts of intravenous fluids are often given during surgery, which may lead to tissue edema and cellular hypoxia. An increase in the cPVS was associated with volume overload and venous congestion, the activation of renin-angiotensin and sympathetic systems, and organ failure [2,3,20,21,22,23]. Indeed, cPVS, a marker of plasma volume contraction and expansion, is gaining attention in the field of cardiovascular disease and surgery [2,24]. On the other hand, a restrictive fluid regimen may lead to fewer complications, as well as shorter hospital stays [25,26]; this approach was adopted by several enhanced recovery after surgery (ERAS) pathways [27,28]. Nevertheless, fluid restriction could increase the risk of hypotension and tissue hypoperfusion, leading to organ dysfunction and failure [23,29,30]. Our findings support the use of cPVS in advanced planning, and further research is warranted to provide more definitive evidence.

In the present study, the median preoperative cPVS value implies that a significant proportion of patients were in a dehydrated state, which was likely due to suboptimal preoperative hydration and/or surgical preparation. The administration of a mean fluid volume of 2.5 L (1.14 L h^−1^) significantly improved the cPVS during surgery, but had no effect on the sublingual microcirculatory flow. Although the distribution of microvascular blood flow is highly heterogeneous and its changes are generally thought to be multifactorial [1,9,10], our findings support a recently published risk-adapted fluid strategy recommending a moderately liberal approach aiming at a positive fluid balance of 1–2 L at the end of surgery [23]. A moderately positive fluid balance could improve the postoperative microcirculatory flow and tissue perfusion, especially when the MAP is maintained between 65 and 120 mmHg [8,11]. However, occult hypovolemia may occur in up to 60% of patients undergoing major surgery [29], and more research is needed to identify the optimal perioperative cPVS target. 

Given the observational nature of this study and the inability to account for all confounders, the findings should be validated in a randomized controlled trial setting. The cPVS may not perfectly reflect the real intravascular volume, while the hydration status was different among patients. However, the cPVS values correlate to those estimated using radiolabeled albumin techniques [16]. Moreover, this study was performed in a single academic department, in which the expertise on cardiovascular dynamics and individualized physiology-guided management has increased significantly over the past four years. Although our data are likely to be representative of contemporary patients undergoing major non-cardiac surgery in other centers, intraoperative management may not be. Thus, large prospective cohort studies are needed to confirm our findings in the future. Finally, we focused on the cPVS derived from the pre- and postoperative laboratory tests, and future studies could investigate its intraoperative trend in an effort to improve the perioperative fluid management.

## 5. Conclusions

The calculated PVS, but not its trend, was associated with an increased risk of organ injury and complications in this cohort. Randomized trials are required to determine whether the relationship between cPVS and the outcome is causal, and therefore, amenable to intervention.

## 6. Perspectives 

The indications of fluid resuscitation—or de-resuscitation—are diverse and aim at ensuring hydration and normovolemia, optimizing venous return, and maintaining perfusion pressure and oxygen transport to tissue. In this concept, the final place of action of fluid therapy is the microcirculation. The latter is the terminal vascular network of systemic circulation consisting of microvessels with diameters of <20 μm through which oxygen is transported to tissues [31]. 

In many patients, clinical signs of impaired organ perfusion may be elusive, especially if the volume status assessment is based on macrocirculatory parameters and non-specific symptoms such as altered mental status and tachypnea, which may be related to causes other than hypo- or hypervolemia. In addition, dynamic assessment methods may not be available, while static filling methods may lead to erroneous assessments. Although some studies showed that the microcirculatory flow may improve after fluid administration and stroke volume increase [32,33], recent evidence suggests that microvascular perfusion is maintained when the MAP ranges between 65 and 120 mmHg [11]. Consequently, circulatory volume imbalances may potentially result in organ injury even in patients with adequate microcirculation, making the estimation of fluid losses or overload challenging [34,35,36,37]. 

The present study confirms the effectiveness of fluid administration in terms of hydration and normovolemia. Furthermore, our observations clearly suggest that imbalances of plasma volume may be related to organ injury irrespective of the microcirculatory flow. This finding deserves caution, considering that the monitoring of microcirculation in the present study was limited to the intraoperative period, while organ injury was evident several hours or days later. Extending the monitoring period beyond the intraoperative time would likely allow us to reveal associations between the cPVS and microcirculatory flow. In reality, every patient is different, and their management cannot be completely protocolized, necessitating an individualized approach to fluid management. The present study provides crucial data and concepts that are necessary to guide the design of a future randomized trial to assess cPVS in several patient populations.

After decades of research on fluid management, it is clear that none of the available indicators, e.g., heart rate, central venous pressure, or lactate, are completely specific and reliable for the assessment of intravascular volume. Moreover, most of the markers are not very sensitive for detecting the changes in the volume status, especially in acutely and critically ill patients with severe physiological impairment, significant changes in the stressed-to-unstressed volume ratio, and mobilization of the splanchnic circulation. Also, the recognition of hypervolemia may be challenging in the anesthetized and mechanically ventilated patient as it can occur independently of physical signs of congestion [38]. Given the above difficulties with correctly assessing the volume status, our study strengthens the evidence suggesting that cPVS may serve as a reliable marker for monitoring the plasma volume and predicting organ injury and complications. The cPVS was validated primarily as a surrogate of intravascular filling in patients with cardiac disease [4,39,40,41,42,43], but the present study suggests that the application of cPVS can be extended beyond cardiovascular outcomes. 

Despite the close relationship between the volume status and tissue perfusion, the microcirculation may not always help in the decision of when to start and stop fluid therapy because of its autoregulatory capacity. However, a lower or—most possibly—a higher-than-normal cPVS may impair convective oxygen transport and diffusion of oxygen [8,44,45]. Changes in the cPVS may imply aggravation of the Fåhræus effect (i.e., the decaying of the relative hematocrit in small vessels as the vessel diameter decreases [46,47,48,49,50]) in patients with shock and/or increased systemic vascular resistance. In dehydrated or hypovolemic patients, an acute decrease in the cPVS may imply further impairment of tissue perfusion, while in hypervolemic patients, an increase in the cPVS may be related with the aggravation of type 2 and/or type 4 microcirculatory alterations [14,51]. 

A deviation of the cPVS from its baseline value may also be related to changes in the red blood cell velocity. A recent clinical study including computational fluid dynamics modeling showed an association between the mean circulatory filling pressure (i.e., a quantitative index of intravascular blood volume modifiable by vascular tone) and the microvascular pressure difference and velocity fields [52]. As oxygen diffusion is related to the pressure gradient and inversely related to the distance between the capillary and the cell [46], the correlation of the cPVS with changes in the red blood cell velocity, and thus, with tissue oxygenation, merits further investigation. 

Fluid therapy remains a contentious area in emergency and critical care medicine, and significant efforts are made to recognize patients who are fluid responsive or fluid tolerant. The cPVS may provide a modality for the administration of the optimal amount of fluid or for optimal de-resuscitation on an individualized basis.

## Figures and Tables

**Table 1:** Demographic and clinical characteristics of patients.

	Males	Females
Age	71 (64–75)	66.5 (61.5–75.2)
Height	173 (170–179)	164 (160–168)
Weight	80 (72.8–88.5)	77.5 (68–85)
BMI	26.2 (24.9–28.4)	29.7 (24–32.7)
ACS-NSQIP	13.2 (8.38–21)	9.75 (4.9–18.3)
POSSUM (morbidity)	32.3 (24.9–51)	31.2 (26–49.6)
POSSUM (mortality)	6.7 (4–11.2)	7.1 (4.7–10)
Modified Frailty Index	3 (1–6)	1 (0–4)
CCI	20.3 (0–30.1)	21 (0–29.5)
cPVS	−7.3 (−14.5–−2)	−7.5 (−14–−1.8)

BMI, body mass index; CCI, comprehensive complication index; cPVS, calculated plasma volume status.

**Table 2:** Correlations between perioperative calculated plasma volume status and sublingual microcirculation.

**Preoperative cPVS**	**Spearman’s rho**	**Adjusted *p*-value**
De Backer score (mm^−1^)	0.011	0.91
Consensus PPV (%)	−0.066	0.91
Consensus PPV (small) (%)	−0.042	0.91
**Postoperative cPVS**	**Spearman’s rho**	**Adjusted *p*-value**
De Backer score (mm^−1^)	0.069	0.63
Consensus PPV (%)	0.049	0.63
Consensus PPV (small) (%)	0.086	0.63

cPVS, calculated plasma volume status; PPV, proportion of perfused vessels.

**Table 3:** Postoperative organ injury and complications.

Complication	N = 100 *
Abdominal hernia	1
Acute coronary syndrome	2
Acute kidney injury	6
Acute pulmonary edema	2
Anemia	24
Hemorrhage	5
Hypotension	6
Ileus	2
Intestinal rupture	1
Liver failure	1
Multiple organ failure	1
Pneumonia	1
Pulmonary embolism	1
Readmission	1
Re-operations	1
Respiratory failure	13
Rhabdomyolysis	1
Sepsis	30
Stroke	3
Surgical wound dehiscence	1
Thrombopenia	1
Urinary infections and pig-tail insertion	1

* Some patients had two or more different types of complications.

## Data Availability

Data can be made available upon request after publication through a collaborative process. Researchers should provide a methodically sound proposal with specific objectives in an approval proposal. Please contact the corresponding authors for additional information.

## Supplementary Materials

The following supporting information can be downloaded at https://www.mdpi.com/article/10.3390/jpm13071085/s1, Table S1: Baseline general blood count and biochemistry; Table S2: Correlations of preoperative calculated PVS with clinical and laboratory variables; Table S3: Correlations of postoperative calculated PVS with clinical and laboratory variables.
