# Supplementary material for: The Relation of Calculated Plasma Volume Status to Sublingual Microcirculatory Blood Flow and Organ Injury
Source: J Pers Med. 2023 Jun 30;13(7):1085. doi: 10.3390/jpm13071085 (PMC10381119; doi:10.3390/jpm13071085)
Supplement: Supplementary file 1 [file jpm-13-01085-s001.zip › Table S1.pdf]

Table S1: Baseline general blood count and biochemistry

	Females	Males
WBC	7150 (5900 – 8650)	7400 (6300 – 8600)
Lymphocytes	1880 (1415 – 2072.5)	1635 (1400 – 2087.5)
Monocytes	540 (400 – 625)	635 (495 – 740)
Neutrophils	4390 (3830 – 5437.5)	4695 (4082.5 – 5870)
Eosinophils	125 (47.5 – 200)	160 (90 – 310)
Basophils	30 (27.5 – 40)	40 (20 – 50)
RBC	4.59 (4.14 – 4.82)	4.63 (4.39 – 4.96)
HGB	12.35 (11.28 – 13.5)	12.65 (11.8 – 14.8)
HCT	37.75 (35.8 – 40.88)	39.4 (37 – 44.7)
MCV	86.95 (83.8 – 90.7)	89.5 (82.75 – 92.2)
MCH	28.6 (27 – 29.42)	29.35 (27.17 – 30.3)
MCHC	32.65 (31.78 – 33)	32.5 (31.9 – 33.1)
RDW	14.2 (13.47 – 15.22)	14.1 (13.2 – 15.17)
PLT	247 (208.75 – 326)	249 (198.75 – 308)
PT	11.4 (11.07 – 12.03)	12.1 (11.4 – 13.35)
INR	0.97 (0.94 – 1.07)	1.03 (0.97 – 1.1)
aPTT	26 (23.5 – 29.77)	27.55 (25.37 – 30.5)
Glucose	98.85 (92.65 – 106.6)	99.5 (91.33 – 122.05)
Urea	33 (23.7 – 43.55)	36.65 (29.15 – 43.1)
Creatinine	0.74 (0.61 – 0.8)	0.85 (0.76 – 1)
CRP	1.2 (0.43 – 2.1)	0.5 (0.23 – 2.1)
SGOT	18.4 (13.9 – 20.7)	17.35 (13.7 – 25.33)
SGPT	17.2 (11.43 – 23.25)	17.2 (11.3 – 23.8)
γGT	14 (11 – 30)	15 (12 – 45.5)
Total bilirubin	0.7 (0.4 – 0.8)	0.7 (0.46 – 0.84)
Direct bilirubin	0.21 (0.1 – 0.3)	0.2 (0.1 – 0.39)
CPK	77.5 (63 – 100)	72 (57.5 – 112.25)
LDH	143 (128.25 – 187)	164 (133 – 199.5)
Total protein	6.88 (6.2 – 7.34)	6.69 (6.1 – 7.29)
Albumin	4.4 (4.18 – 4.49)	4.18 (3.87 – 4.42)
Alkaline phosphatase	61 (45 – 74.25)	71 (52.75 – 89)

	Females	Males
Amylase	53 (39 – 66)	57 (43.75 – 87)
Calcium	8.1 (7.9 – 8.8)	8.1 (7.9 – 8.75)
Potassium	4.42 (4.05 – 4.8)	4.5 (4.24 – 4.73)
Sodium	139.55 (138 – 140.95)	140 (137.38 – 141.83)
