# Supplementary material for: The Relation of Calculated Plasma Volume Status to Sublingual Microcirculatory Blood Flow and Organ Injury
Source: J Pers Med. 2023 Jun 30;13(7):1085. doi: 10.3390/jpm13071085 (PMC10381119; doi:10.3390/jpm13071085)
Supplement: Supplementary file 1 [file jpm-13-01085-s001.zip › Table S2.pdf]

Table S2: Correlations of preoperative calculated PVS with clinical and laboratory variables

	**Spearman's rho**	**Adjusted p-value**
Age	0.091	0.609
Weight	-0.560	**<0.001**
Height	-0.159	0.276
BMI	-0.508	**<0.001**
Modified Frailty Index	-0.033	0.823
POSSUM (morbidity)	0.422	**<0.001**
POSSUM (mortality)	0.418	**<0.001**
ACS_NSQIP	0.108	0.521
APACHE II	0.001	0.994
SOFA	-0.098	0.565
WBC	-0.089	0.609
Lymphocytes	-0.281	**0.023**
Monocytes	0.046	0.796
Neutrophils	-0.016	0.890
Eosinophils	-0.044	0.796
Basophils	-0.112	0.507
RBC	-0.504	**<0.001**
HGB	-0.838	**<0.001**
HCT	-0.859	**<0.001**
MCV	-0.102	0.551
MCH	-0.086	0.609
MCHC	-0.154	0.279
RDW	0.225	0.085
PLT	0.083	0.609
PT	0.332	**0.004**
INR	0.333	**0.004**
APTT	0.230	0.085
Glucose	0.024	0.861
Urea	0.049	0.796
Creatinine	-0.048	0.796
CRP	0.191	0.166
SGOT	0.129	0.413
SGPT	-0.042	0.796
γGT	0.156	0.279
Total bilirubin	-0.083	0.609
Direct bilirubin	-0.058	0.758

	Spearman's rho	Adjusted p-value
CPK	-0.036	0.813
LDH	0.040	0.797
Total protein	-0.223	0.085
Albumin	-0.226	0.085
Alkaline phosphatase	0.068	0.697
Amylase	0.017	0.890
Calcium	0.132	0.401
Potassium	-0.119	0.464
Sodium	-0.057	0.758
