# Supplementary material for: The Relation of Calculated Plasma Volume Status to Sublingual Microcirculatory Blood Flow and Organ Injury
Source: J Pers Med. 2023 Jun 30;13(7):1085. doi: 10.3390/jpm13071085 (PMC10381119; doi:10.3390/jpm13071085)
Supplement: Supplementary file 1 [file jpm-13-01085-s001.zip › Table S3.pdf]

Table S3: Correlations of postoperative calculated PVS with clinical and laboratory variables

	Spearman's rho	Adjusted p-value
Age	0.270	**0.027**
Weight	-0.520	**<0.001**
Height	0.004	0.996
BMI	-0.554	**<0.001**
Modified Frailty Index	0.069	0.642
POSSUM (morbidity)	0.347	**0.004**
POSSUM (mortality)	0.340	**0.004**
ACS_NSQIP	0.230	0.063
APACHE II	0.104	0.472
SOFA	0.001	0.996
WBC	-0.137	0.347
Lymphocytes	-0.258	0.034
Monocytes	0.078	0.625
Neutrophils	-0.077	0.625
Eosinophils	-0.015	0.979
Basophils	-0.141	0.342
RBC	-0.318	**0.008**
HGB	-0.745	**<0.001**
HCT	-0.813	**<0.001**
MCV	-0.314	**0.008**
MCH	-0.168	0.210
MCHC	-0.024	0.961
RDW	0.178	0.182
PLT	-0.206	0.101
PT	-0.006	0.996
INR	0.047	0.813
APTT	0.258	**0.034**
Glucose	-0.123	0.408
Urea	-0.116	0.418
Creatinine	-0.014	0.979
CRP	0.246	**0.046**
SGOT	0.072	0.642
SGPT	0.001	0.996

	Spearman's rho	Adjusted p-value
γGT	-0.033	0.921
Total bilirubin	0.070	0.642
Direct bilirubin	0.109	0.451
CPK	-0.002	0.996
LDH	-0.120	0.408
Total protein	-0.321	**0.008**
Albumin	-0.294	**0.014**
Alkaline phosphatase	-0.240	0.051
Amylase	-0.028	0.939
Calcium	0.119	0.408
Potassium	-0.272	**0.027**
Sodium	-0.136	0.347
